# Women in Saudi secondary school EFL textbooks: a critical study of women’s empowerment as enshrined in the Saudi Vision 2030

**DOI:** 10.3389/fsoc.2024.1307623

**Published:** 2024-03-25

**Authors:** Muneer Hezam Alqahtani

**Affiliations:** College of Education, King Faisal University, Al-Ahsa, Saudi Arabia

**Keywords:** Saudi Vision 2030, secondary school books, women’s empowerment, EFL textbooks, pictorial representation

## Abstract

Since the launch of the Saudi Vision 2030, Saudi Arabia has undergone significant economic and social reforms in order to move away from the country’s reliance on oil and toward a more diverse and sustainable economy. One of the important chapters in this endeavour is the empowerment of Saudi women whereby they are to be given opportunities equal to men. Against this new paradigm, this study investigates whether Vision 2030’s transformative efforts surrounding women’s empowerment are reflected in the country’s EFL textbooks, or whether the traditional representation of Saudi women has remained unchanged. The analytical focal point is the pictorial representation of Saudi women in six textbooks which form part of the Mega Goal series, and which are used to teach English in Saudi secondary schools. The analysis examined the representations of females from three different angles: the percentage of appearances in the textbooks from the total human pictorials; the social roles and occupations depicted, and the activities that they are engaged in. Analysis along all three of these angles revealed that there is a remarkable imbalance between the depiction of men and women in these textbooks, in favour of men. The study concludes that the Mega Goal series’ EFL textbooks fall short of providing a realistic representation of Saudi women and fails to include representative depictions of women who, like their male counterparts, have occupied senior roles and prestigious positions in the country. This conclusion points to the need to include such representation in EFL textbooks, so that the role of women as envisaged in Saudi’s Vision 2030 complies with the Vision’s determination to provide equal opportunities for both men and women.

## Introduction

The plummet in oil prices during 2014 had a widespread impact on oil-producing nations, including Saudi Arabia. By 2015, the country faced a substantial USD 100 billion budget deficit (Ministry of Finance, 2020), prompting a drastic 26% reduction in public expenditure (Ministry of Finance, 2020). This financial crisis revealed the perils of relying solely on oil revenues for the nation’s income. Consequently, the government recognized the urgent need to diversify its resources beyond oil (Alqahtani and Albidewi, 2022). This realization culminated in the introduction of an ambitious national development plan, Vision 2030, in spring 2016. The primary objective of Vision 2030 was to transition the country’s economy and society away from its traditional dependence on oil revenue, fostering a more diversified economy and a globally engaged society (Alqahtani, 2022, p. 580). The Vision outlined three interdependent pillars: a robust economy, a dynamic society, and a forward-thinking nation. Each pillar encompassed several strategic objectives and implementation programs crucial to realizing the goals of Vision 2030. However, the successful execution of this Vision hinges on broad participation across all sectors and by all individuals within the country. Hence, Vision 2030 places significant emphasis on facilitating equal opportunities for women in the labor market, acknowledging women as valuable assets (Saudi Vision 2030, 2016, p. 37) and advocating for their active inclusion (Al-Asmari, 2008; Korany, 2010; Aluwaisheg, 2013; Alfarran, 2016). The concerted efforts to provide women with equal footing in the labor market yielded unexpectedly positive outcomes. Women’s participation in the workforce not only exceeded the initial target of reaching 30% by 2030 but had already surpassed that mark, standing at 31.8% by the end of 2020, as reported by The Ministry of Human Resources and Social Development (2023). Impressively, women held 30% of senior positions, and their representation in the civil service stood at 41.02%. Demonstrating remarkable competency, Saudi women showcased leadership skills across diverse roles in government, serving as ambassadors, ministers, directors, consultants, and even astronauts since the inception of the Saudi Vision 2030.

Despite strides towards equality, traditional depictions of Saudi women as subservient to men persist within English as a Foreign Language (EFL) textbooks. This (mis) representation finds roots in the educational framework that molds perceptions about women (Eslami et al., 2015). Sulaimani (2017) contends that educational discourse significantly influences one’s identity, positively or negatively. Furthermore, Romera (2015, p. 206) asserts that educational institutions shape gender identities, constructing societal notions of masculinity and femininity. The discourse prevalent in textbooks serves as a potent instrument that molds social power dynamics and reinforces gender stereotypes (Kobia, 2009; Sulaimani, 2017). Particularly in EFL education, textbooks hold substantial sway, acting as conduits for learners to assimilate the culture and values of the target language, notably in lecture-oriented settings prevalent in countries like Saudi Arabia (Bawazeer, 2015).

### Literature review and theoretical background

As per the United Nations, there are seven principles that signal women’s empowerment. These are: establishing corporate leadership that promotes gender equality, ensuring equal treatment and non-discrimination in the workplace, guaranteeing worker health and safety, promoting education and professional development for women, supporting women’s entrepreneurship and empowerment policies, advocating for equality through community initiatives, and measuring and publicly reporting progress towards gender equality (The United Nations Entity for Gender Equality and the Empowerment of Women, 2018). Literature on women’s empowerment is varied and rich, with studies having been conducted in different environments. Even so, a gap exists in the theoretical dimensions of what the term entails (Grown et al., 2005; Cueva Beteta, 2006; Gholipour et al., 2010; Upadhyay et al., 2014). The reason for this is that this issue is highly contextualized as its definition and scope fall in the purview of the socio-cultural context which is a factor of religious and social heritage and practices (Alkhaled and Berglund, 2018; Cinar and Kose, 2018). For instance, Kabeer (1999) identified three stages of empowerment, i.e., resources, agency, and achievement. Resources refer to what the society can offer in terms of material, human, and social expectations and allocations, whereas agency is related to women’s independence and achievement displays the results of the process of empowerment.

One of the major settings where the empowerment of women Jinia, (2016) could be visible and an indicator of their status in a given context is how they are represented in textbooks. Images and pictures offer a universal language that carry the same literacy elements as of verbal language (Olshansky, 2008). For example, “just as you can analyze how the first paragraphs of text establish a sense of setting and mood at the beginning of a story, you can also analyze the “setting picture” in a quality picture book, noting how it establishes a sense of setting and mood through the use of visual elements.” (Olshansky, 2008, p. xii). Therefore, images convey powerful meanings and have been used alongside written texts in order to facilitate learners’ comprehension (Basal et al., 2016).

When it comes to the representation of gender in EFL textbooks, the issue was studied extensively back in the 1970s and 1980s (Sunderland, 2000; Pakuta et al., 2014; Tahriri and Pouran, 2014). At that time, most studies depicted women as passive, dependent, or weak, whilst men were depicted as independent, strong, and active (Sulaimani, 2017). Despite this extensive early research, gender representation in EFL textbooks remains under-researched in Arab and Islamic countries, including in Saudi Arabia (Sunderland, 2000; Sulaimani, 2017). One study that investigated the (mis) representation of women in EFL textbooks in the Arab countries was conducted by Al-Taweel (2005). She examined the EFL textbooks prescribed at high schools in Jordan and explored the presence of both men and women in the textbook and the nature of depiction. The results showed that men were represented in 70% of the textbooks whilst women were represented in only 25%. She also found that “Most of the female references are presented in contexts that are specifically related to women, with partial dependence on men, while most male references were used as Pseudo-masculine references” (Al-Taweel, 2005, p. iv). Al-Taweel (2005) also concluded that more than half of the teachers who participated in the study were unaware of such (mis) representation of women in the EFL textbooks that they were using in their teaching. Another study investigating the (mis) representation of women in EFL textbooks in Arab countries was carried out by Qatar National Research between the years 2009 and 2011. The study examined the stereotyping of different gender roles in Qatari schools at different levels (Ismail et al., 2011; Yasin et al., 2012). Both quantitative and qualitative methods were used to conduct the study and the results indicated that men were more visible than women in the EFL textbooks. Moreover, in terms of their jobs, men were depicted in a variety of traditionally male roles, whilst women were depicted as having very limited choices. The study demonstrates the continuation of the (mis) representation of women in EFL textbooks in Arab and Islamic countries.

In the specific context of Saudi Arabia, only four studies have focused on the (mis) representation of women in EFL textbooks (Al-Jumiah, 2016; Sulaimani, 2017; Aljuaythin, 2018; Almghams, 2020). In the first study, Al-Jumiah (2016) explored how social power and gender ideology were represented in two high school EFL textbooks in use in Saudi Arabia. The textbooks that he investigated were called *Flying High Saudi Arabia 3* and *Flying High Saudi Arabia 4*. Both textbooks were published by Macmillan in 2013. His findings show that the hidden and apparent discourses in the EFL textbooks continue to perpetuate and reinforce gender ideologies in which men seem to possess more supremacy, dominance, and power, whilst women appear to be subordinate and marginalised.

The second study on the (mis) representation of women in EFL textbooks in Saudi Arabia was carried out by Sulaimani (2017). She scrutinised gender representation in EFL textbooks at a Saudi state university, rather than school textbooks. The textbooks that she analysed were the North Star Middle Eastern Edition series, published by Pearson Education and were used by the university where she conducted her study between the years 2010–2013. The second series that Sulaimani (2017) analysed were the New Headway Plus Special Edition series published by Oxford University Press and were used at the state university between 2013 and 2015. Finally, she analysed a series called English Unlimited Special Edition series published by Cambridge University Press and were being used by the same university at the time of conducting her study. She investigated gender frequencies conversations in three dimensions: gender relations, subject positions, and contents. The results show that there is a clear bias in the way both men and women were represented. Women were underrepresented and were excluded from half of the books’ units. Moreover, though both men and women were positioned in the same subjects and contents, the male characters remarkably outnumbered the females.

The third study was conducted by Aljuaythin (2018) who examined two EFL textbooks from the series *Smart Class 5* and *Smart Class 6* published by MM Publications in 2016; these are used to teach English at high school level. The study analysed four aspects of the textbooks: the frequencies with which men and women occurred; the activities that they are engaged in; the pictorial representations of the two genders; and the social roles that are associated with men and women. The analysis shows that the representation of men and women in the textbooks was unbalanced in favour of men. Women were marginalised and were reduced to traditional roles. She concludes that “underrepresentation of women could create a false reality surrounding perceptions of women and hinder the process of ensuring equality to all humans” (Aljuaythin, 2018, p. 151).

More recently, Almghams (2020) studied the gender representation in the second edition of *Family and Friends KSA*, which was published by Oxford University Press in 2017. This textbook is used predominantly in private schools in Saudi Arabia. Her findings show that women are under-represented in terms of their presence in the textbooks and that men were more prevalent in long passages and conversations. Moreover, she states that “there is an overall higher tendency for females to be referenced in sentences before males” (Almghams, 2020, p. 1). Further, although the textbooks did at times show women’s empowerment by, for example, demonstrating the equal distribution of household responsibilities between men and women and by indicating that women’s activities were not restricted to being passive or staying indoors, men were still represented in a wider range of jobs and, compared to women, they were shown to occupy more of the senior positions. They also had a wider range of spare time and leisure activities.

Despite the attempts of the above studies to investigate the (mis) representation of women in EFL textbooks used in Saudi Arabian EFL teaching, they fall short in addressing the issue which can be attributed to their being conducted before the nation was given a comprehensive developmental plan. For example, Al-Jumiah’s (2016) study was conducted before the launch of the Saudi Vision 2030, which advocates the equality between men and women in Saudi Arabia. Therefore, the study investigated textbooks during times when the equality between men and women in Saudi Arabia had not been strongly supported by the state as of today. Similarly, Sulaimani’s (2017) study investigated textbooks that were used at the state university where the study was conducted in the years between 2010 and 2017, which is either before the launch of the Saudi 2030 Vision or shortly after. The same reservation applies to Aljuaythin (2018) and Almghams (2020) because the textbooks they examined were published in 2016 and 2017 respectively, at a time when the efforts towards women’s empowerment embodied in the Saudi 2030 Vision had not yet taken form.

In the current times, however, global geopolitical dynamics have acted as the proverbial shot in the arm. Hence, this study seeks to examine the representation of women in the most recent (2023) editions of Saudi EFL textbooks prescribed in secondary schools. The emphasis on the current year is pivotal for this investigation for two primary reasons. Firstly, prior studies (such as, Alghamdi, 2014; Al-Shahrani, 2016; Quamar, 2016) addressing this issue were conducted pre-dating the launch of the Saudi Vision 2030 (announced on 25 April, 2016) or shortly thereafter. Consequently, their findings outlined evident (mis) representations of Saudi women in EFL textbooks, a topic elaborated on in subsequent sections of this research. Secondly, 2023 signifies the midpoint since the initiation of the Saudi Vision 2030 in 2016, a juncture marked by the evaluation of achieved objectives and the potential need for course correction. Hence, it becomes pertinent to reassess the portrayal of Saudi women in EFL textbooks, gauging any advancements or persisting imbalances.

### Research questions

To fill the knowledge gap, this study sets out to review the (mis) representation of women in EFL textbooks in Saudi Arabia in light of the government’s efforts to empower women and provide them with opportunities equal to men as envisaged in Vision 2030. Hence, the study endeavours to answer the following research questions:

1. How much (in comparison to men in terms of percentages of the total human pictorials) are women represented in the current EFL Mega Goal series textbooks in Saudi Arabia, and what is the nature of this representation?
2. Has the Saudi Vision 2030’s attempts to empower Saudi women been reflected in EFL Mega Goal series textbooks?

## Methodology

### Material

In order to answer these research questions, this study reviews six EFL textbooks from the Mega Goal series published by McGraw-Hill Education in 2023. This is the newest edition of the series, and EFL teachers use these texts in their teaching in secondary schools in Saudi Arabia. The focus will be on: *Mega Goal 1* and *Mega Goal 2* (prescribed at the first grade at high school), *Mega Goal 3* and *Mega Goal 4* (used for teaching at the second grade) and *Mega Goal 5* and *Mega Goal 6* (meant for the third grade).

### Data collection

This study focused on the frequencies with which pictures of men and women appear in each of the textbooks mentioned above. Further, the pictures were analysed in terms of the social roles of men and women as well as the activities they are shown to be engaged in. The reason for focusing on images rather than other forms of communication in the texts is based on previous studies that established that pictures and images offer a universal language Kress and van Leeuwen (2006) (Olshansky, 2008), convey meanings (Basal et al., 2016) and are available in almost every textbook (Elmiana, 2019). When it comes to EFL textbooks, images provide both teachers and learners with useful tools to enhance language learning in the classroom (Jahangard, 2007). Moreover, images are tools for communication (Moghtadi, 2012) and are powerful elements that shape learners’ worldviews (Canning-Wilson, 1999). Therefore, with these theoretical assumptions, this study focuses solely on investigating the frequencies with which pictures of men and women appear in the EFL textbooks and the associations that these images may entail (Fakher, 2011).

### Data analysis

This study replicates Elmiana (2019) in analysis of the visual images in EFL textbooks in Indonesia, based on the three different modes of visuals: the representational mode; the interactive mode; and the compositional mode.

The representational mode involves the description of the participants, inanimate or animate, the activities represented by the participants and the settings within which the representation is developed. The interactive mode is built by the viewer from the way the visual image addresses its potential viewers in interactional terms. The compositional mode focuses on the aspects of page layout that determine whether the visual and verbal elements have coherence in the texts (Elmiana, 2019, p. 3).

Elmiana (2019) used the three modes presented above to investigate visual images in EFL textbooks in Indonesia from different perspectives. Although different themes emerged from her analysis, the representation of men and women was a constant theme in her study. She investigated the theme from three different angles: the percentage of appearance of men versus women in the textbooks; their social roles (occupations); and the activities that they are shown to be engaged in. In the current study, the data was also analysed using these three dimensions (percentages, occupations and activities) that Elmiana (2019) employed as the current study is intended as a replication of Elmiana in the Saudi context.

## Findings

**Figure 1:**
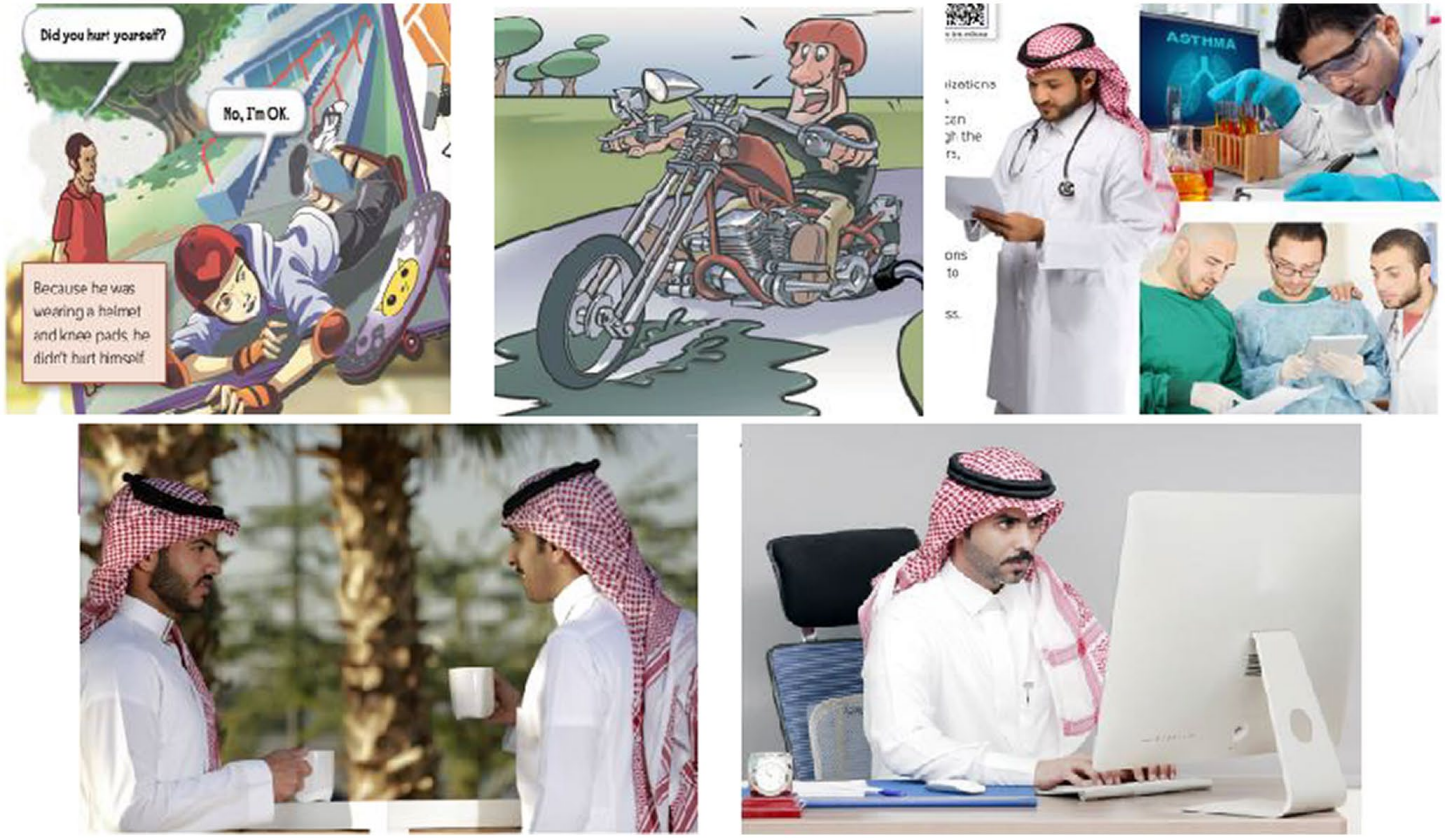
The wide varieties of activities and occupations of men as depicted in *Mega Goal 1* and *Mega Goal 2* textbooks.

**Figure 2:**
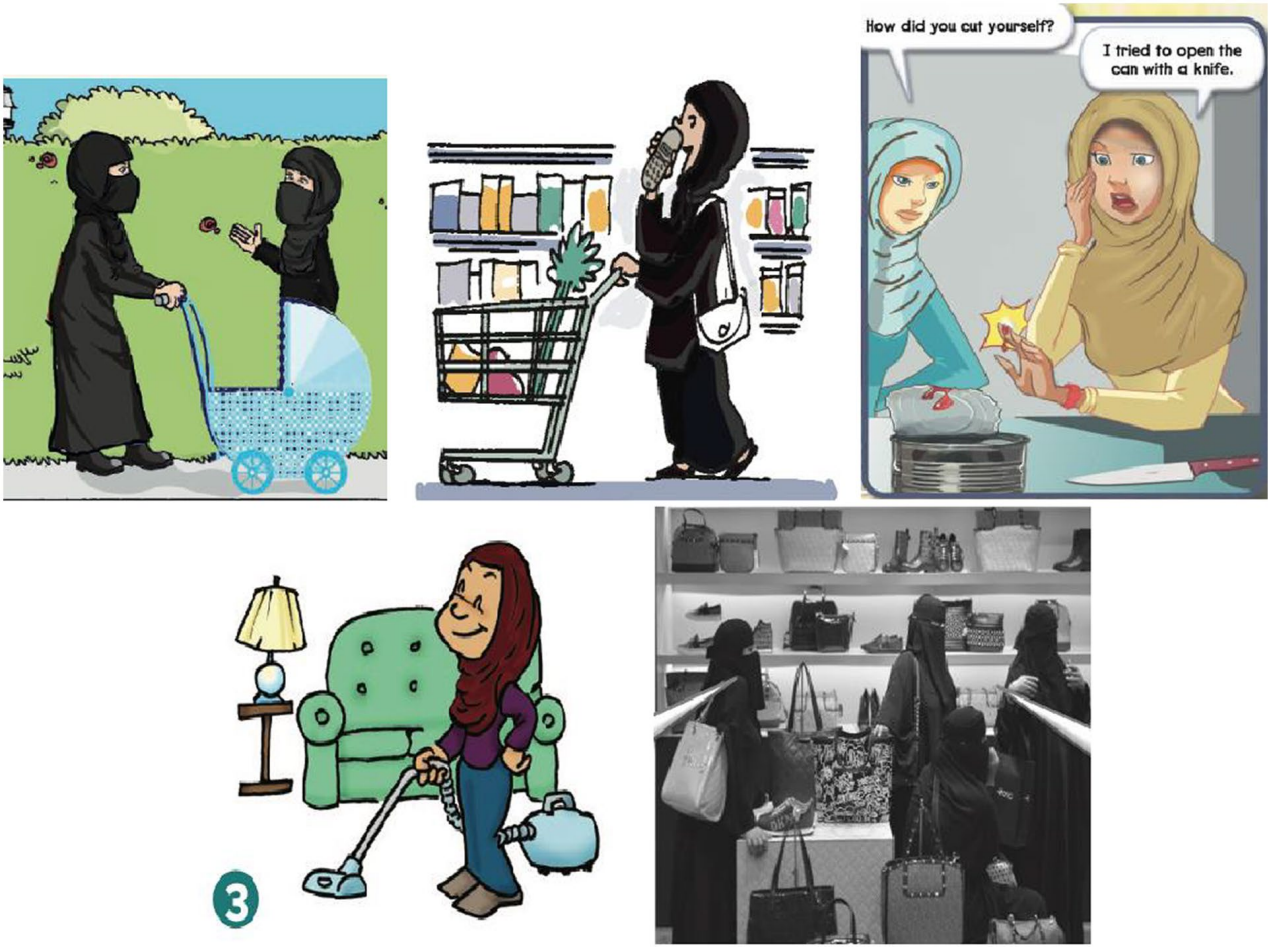
The small varieties of activities and occupations of women as depicted in *Mega Goal 1* and *Mega Goal 2* textbooks.

The findings of this study demonstrate two forms of unequal representations of both men and women in the Mega Goal textbooks: the frequency of their appearances and the varieties of occupations and activities that they are engaged in. First, when it comes to frequency of appearances, women’s appearances were significantly outnumbered by those of men in the *Mega Goal 1* and *Mega Goal 2* textbooks used when teaching the first grade in secondary schools (see Tables 1, 2). Moreover, the varieties of jobs and activities that both men and women were engaged in differed noticeably. Men had more occupational variety (e.g., footballers, entrepreneurs, scientists, pilots… etc.), while women were reduced to more stereotypically traditional occupations (mothers, housewives, housekeepers). Moreover, men were presented as engaging in a wider selection of activities that ranged from playing dangerous sports to chatting to friends (see Figure 1). Women, on the other hand, were presented as being involved in a much smaller selection of activities that mainly revolved around children and family, for example cooking, pushing a stroller, or cleaning the house (see Figure 2).

**Table 1:** Percentage of contribution in the total human pictorials, social roles (occupations) and activities of men and women in the *Mega Goal 1* textbook.

Gender	Percentage contribution in the total human pictorials	Social (occupation)	Activities
Men	91.3%	Footballer Entrepreneur Self-learner Tennis player Car sculptor Food scientist Animation designer Car driver Shopkeeper TV reporter Archaeologist Teacher Horse rider Electrician Paramedic Pilot Surgeon Motorcyclist	Playing sports Making phone calls Driving Researching Reporting on TV Excavating Teaching Going to the cinema Riding horses Skiing Fixing items Training at a gym
Women	8.7%	Mother Housekeeper Housewife	Pushing a stroller Shopping Chatting on a phone Vacuum-cleaning Cooking

**Table 2:** Percentage of contribution in the total human pictorials, social roles (occupations) and activities of men and women in the *Mega Goal 2* textbook.

Gender	Percentage of contribution in the total human pictorials	Social roles (occupations)	Activities
Men	98%	Adventurer Technology enthusiast Nature lover Office employee Father and son student Police officer Self-defence coach Shopkeeper Football spectator Traveller Explorer TV host Chef/Cook Camel rider Writer Ship’s captain	Chatting to friends Discussing work Playing video games Watching football Travelling by plane Hosting a TV show Carrying heavy items
Women	2%	Mother Housekeeper Housewife	Reading a to-do list

As depicted in Table 1, an analysis of the picture content shows that out of the total number of human pictorials, 91.3% were those of men as against 8.7% of women in the *Mega Goal 1* textbook, the grade 1 book in the secondary school stage. This is an important fact to note as it conveys the unwritten message that women are either (i) not as active as men in the socio-economic sphere or (ii) their contribution need not be mentioned or acknowledged. The only roles that they are assigned are, in fact, those of indirect economic contribution as mother, housewife, and housekeeper. This carries a subtle but unmistakable reflection of the early conditioning that Saudi schoolchildren are exposed to about the status of the Saudi women. With an undertone of socially preference, this sets certain gender ideals or roles for the young minds to ape and realize in their adult lives. The depiction of women in the pictorials in the Grade 2 book further decreased as the following data shows.

In the grade 2 book, the depiction of women in the human pictorials further dropped to 2% while the roles assigned to them remained the same as mother, housekeeper, housewife. Even the activities that the women are shown to engage in are reduced to ‘Reading a to-do list’, there being no role assigned to them in the economic sphere. In other words, their professional or economic emancipation (to increase female participation in the workforce to 30% as against 15% up until 2018) is not visible in the text book. They are portrayed as engaging in dormant or passive activities such as ‘Reading a to-do list’.

**Figure 3:**
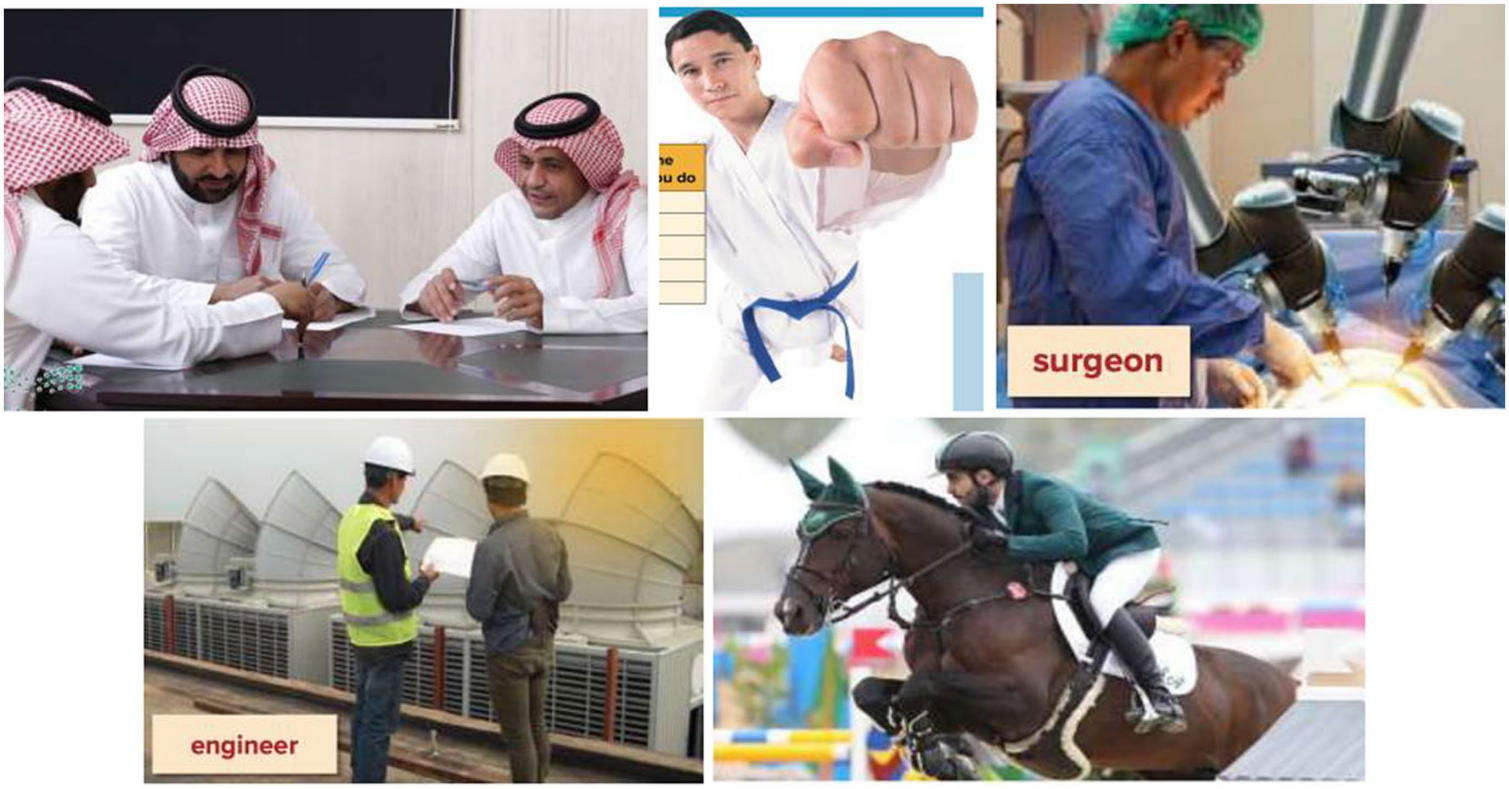
The wide varieties of activities and occupations of men as depicted in *Mega Goal 3* and *Mega Goal 4* textbooks.

**Figure 4:**
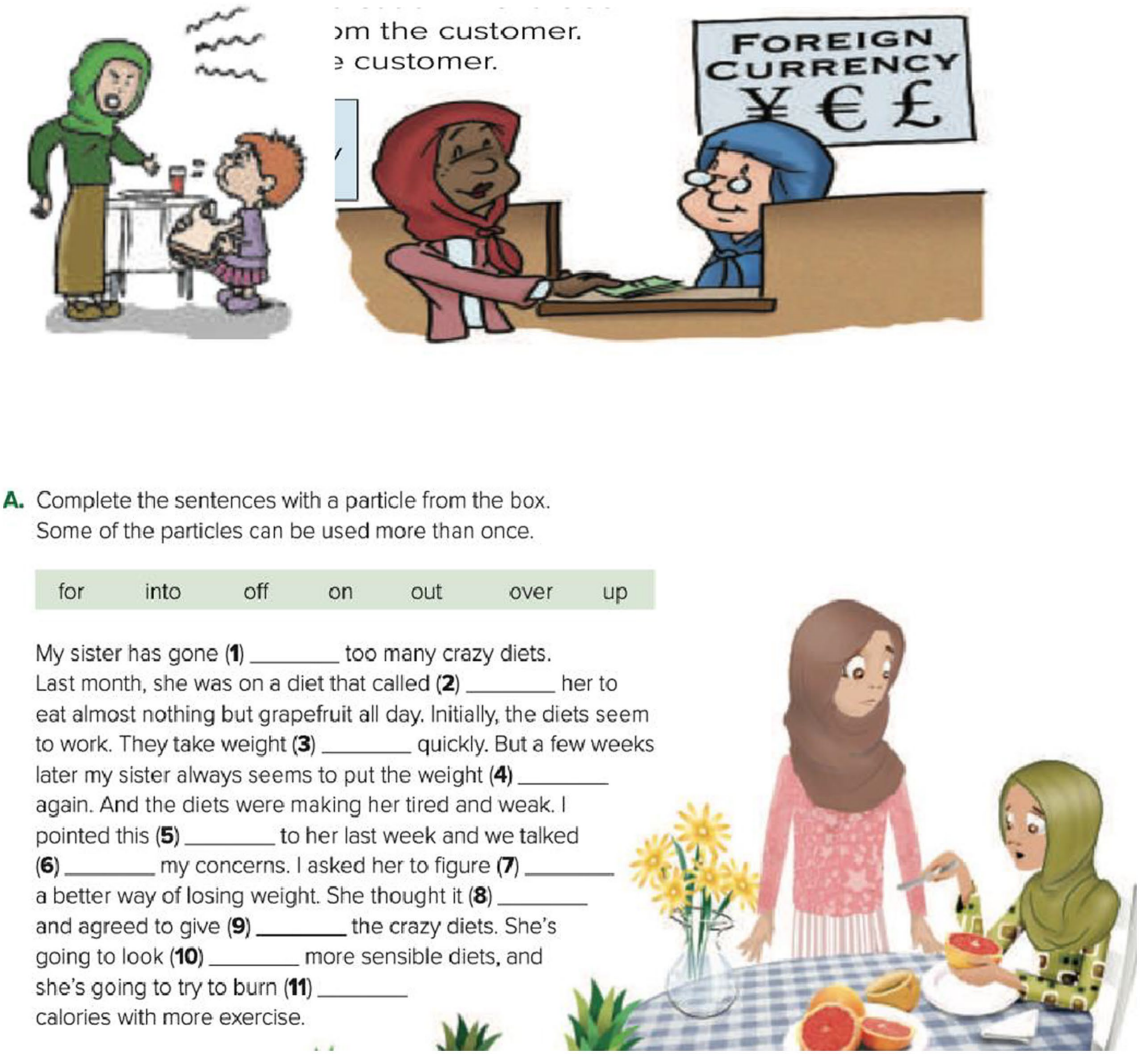
The small varieties of activities and occupations of women as depicted in *Mega Goal 3* and *Mega Goal 4* textbooks.

The same results were found in the *Mega Goal 3* and *Mega Goal 4* textbooks. The percentage of images of men vastly surpassed those of women (see Tables 3, 4). Moreover, men were presented with a wider range of occupations compared to women who were reduced in the image of shoppers. Furthermore, when it comes to the activities that they were engaged in, not only did men have a wider range of activities but also their activities were linked to masculinity and risk-taking (e.g., police officer, horse rider, hockey player) or superiority such as being in a business meeting (see Figure 3). This depiction points towards the greater freedom that men enjoy in the society as well as better acceptance of their choices though the same choices are nowhere depicted with women characters. This clearly shows a high degree of gendered role assignment with better, dominant, and power-wielding roles (physical and mental) revolving around men, whether directly or indirectly, portraying women as demure, weak, and inactive. In other words, women were depicted as engaging in more passive, socially assigned activities such as feeding a child and attending a class. In fact, one paragraph with an image described a woman as going on a “crazy diet,” thus drawing a negative picture of women in comparison to the more ‘athletic’ and ‘competitive’ picturization of men (see Figure 4). Analyzed from a multimodal point of view, the poor depiction is not balanced by any textual content that could convey other ‘empowering’ facts such as the content of the ‘to do list’ which could show other active, economic participation of the woman character.

**Table 3:** Percentages of contribution in the total human pictorials, social roles (occupations) and activities of men and women depicted in the *Mega Goal 3* textbook.

Gender	Percentage of contribution in the total human pictorials	Social roles (occupations)	Activities
Men	96%	Office worker Police officer Self-defence instructor Traveller TV host Cameraman Race car driver Zoologist Engineer Surgeon Nurse Air traffic controller Dentist Mechanic Postman Maths teacher Gardener	Discussing work Surfing the net Interviewing hosts Researching Performing operations Using a phone Interviewing guests on TV
Women	4%	Shopper	Using a foreign currency exchange service

**Table 4:** Percentages of contribution in the total human pictorials, social roles (occupations) and activities of men and women depicted in the *Mega Goal 4* textbook.

Gender	Percentage of contribution in the total human pictorials	Social roles (occupation)	Activities
Men	95.5%	Businessman Falcon trainer Office worker Horse rider Olympic athlete Skater Hockey player Footballer Student Bowler English teacher Scientist Surgeon Lab operator	Surfing the web Picnicking in the desert Discussing work Playing hockey Playing football Attending class Playing bowls
Women	4.5%	Mother Student	Feeding a child Attending a class Going on a “crazy diet”

In the grade 3 book, the percentage of women’s pictures in the total human pictorials was still hovering at a mere 4% which is negligible compared to that of men at 96%, their ‘occupation’ is shown as that of ‘shopper’ which highlights the comic and caricatured portrayal of women in much of the literature of the world as being fond of shopping while men are shown in either the physically or mentally challenging occupations such as self-defence instructor, or mathematics teacher.

The grade 4 book again gave only 4.5% of the pictorial space to women as against 95.5% given to men, the occupational roles are once again limited to the domestic sphere with the exception of portrayal as a student. However, in the grade 5 and 6 books the pictorial depiction of women saw a larger share at 19 and 13.5% respectively, though the occupational status still revolved around those of homemaker, teacher, or paralegal, while the activities showed them in stereotypical engagements such as chatting and drinking coffee.

**Figure 5:**
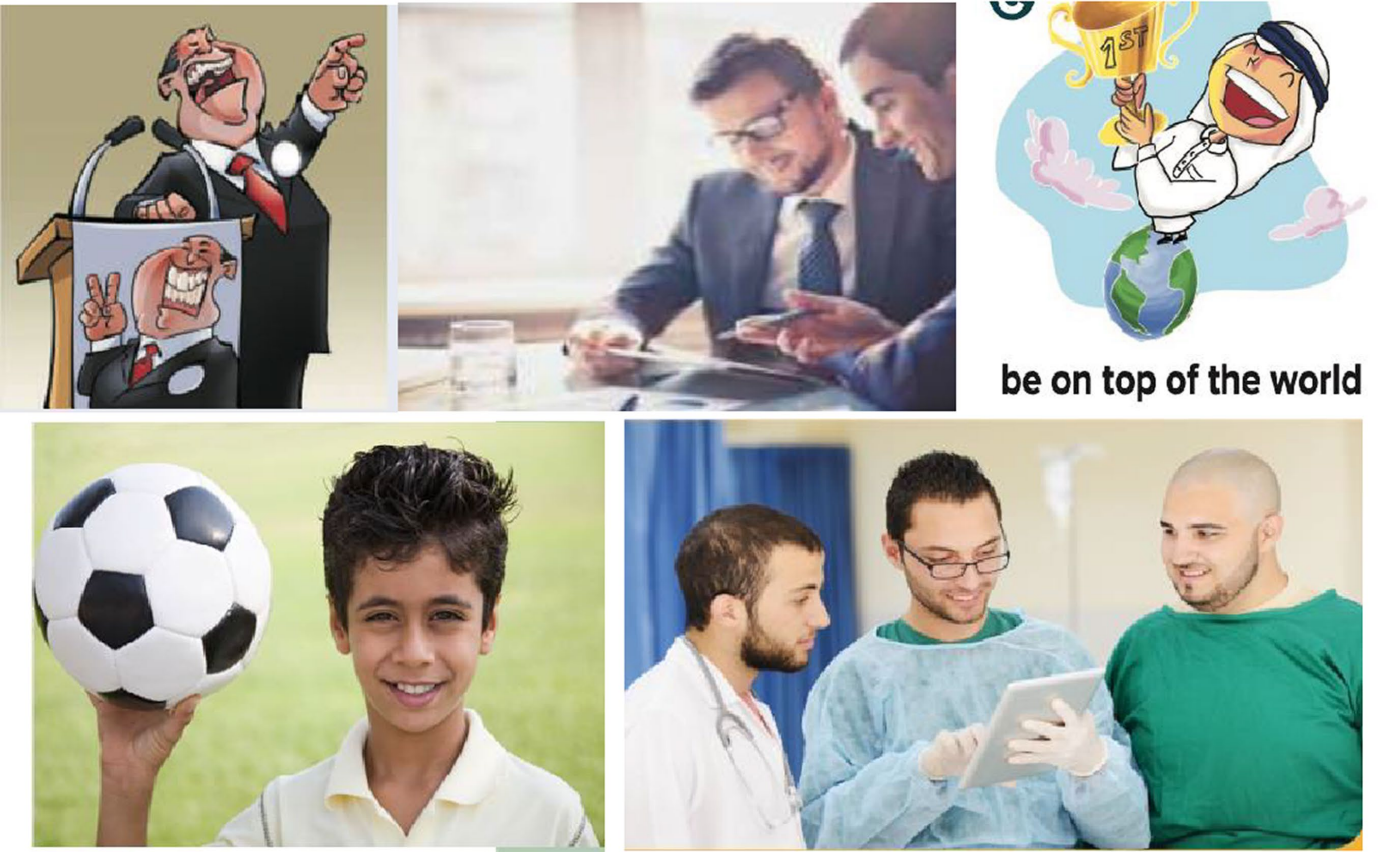
The wide varieties of activities and occupations of men as depicted in *Mega Goal 5* and *Mega Goal 6* textbooks.

**Figure 6:**
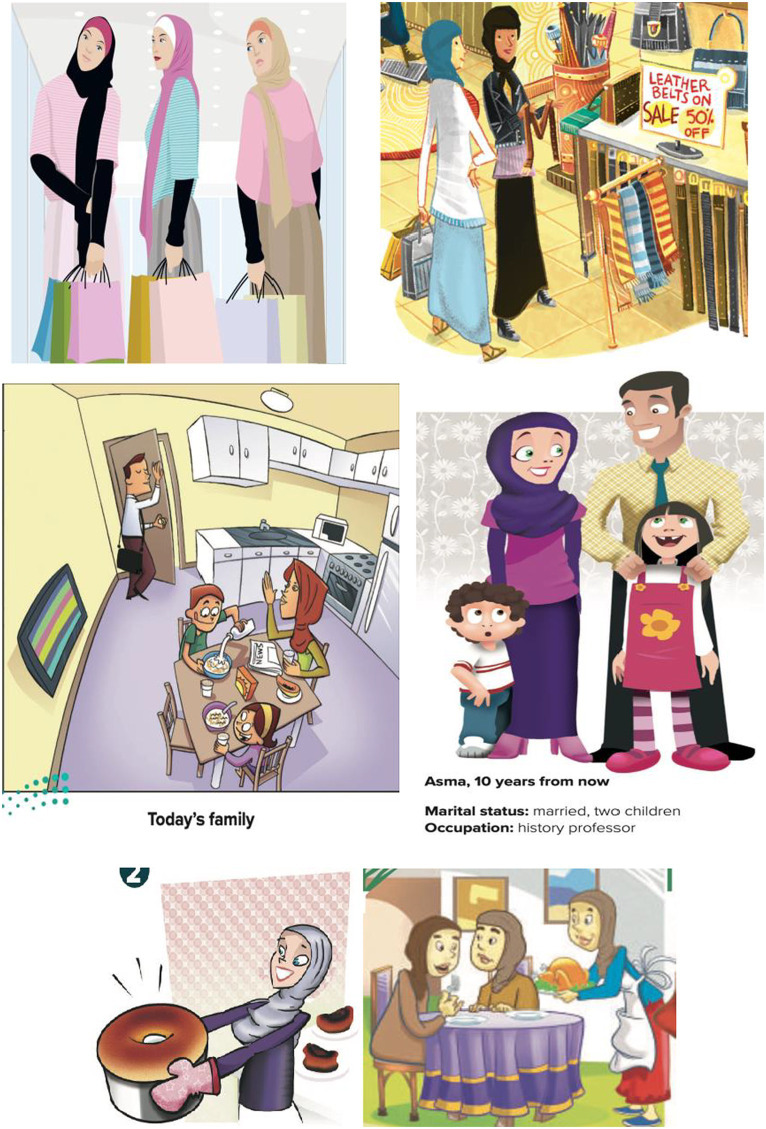
A slightly larger varieties of activities and occupations of women as depicted in *Mega Goal 5* and *Mega Goal 6* textbooks.

Although the *Mega Goal 5* and *Mega Goal 6* textbooks displayed similar imbalances between men and women when it comes to their representation (see data in Tables 5, 6), the percentages were slightly higher than those for the other four textbooks. In addition, in these two textbooks women were presented in a wider range of professions (e.g., a history professor, a nurse) in contrast to the previous textbooks. It is not clear whether the slightly larger percentage of women’s appearances and their wider range of activities in the *Mega Goal 5* and *Mega Goal 6* textbooks had anything to do with the learners’ higher grade level. Nevertheless, the depictions of women in a wider variety of roles, occupations and activities was still small and marginal in comparison to the images and the activities and occupations that men were shown as engaging in (see Figures 5, 6).

**Table 5:** Percentages of contribution in the total human pictorials, social roles (occupations) and activities of men and women depicted in the *Mega Goal 5* textbook.

Gender	Percentage of contribution in the total human pictorials	Social roles (occupation)	Activities
Men	81%	Doctor Entrepreneur Scientist Engineer Surgeon Religious clerk Father Detective Footballer Businessman Postman Lecturer	Watching TV Using an ATM Studying abroad Cleaning the house Working on a laptop Chatting on the phone Going to work Watching basketball on TV Driving a car Using a tablet Being busy at a business meeting
Women	19%	Nurse Mother Housewife Student History professor	Feeding a child Shopping Chatting Cooking

**Table 6:** Percentages of contribution in the total human pictorials, social roles (occupation) and activities of men and women depicted in the *Mega Goal 6* textbook.

Gender	Percentage of contribution in the total human pictorials	Social roles (occupation)	Activities
Men	86.5%	Virologist Chef Father Hairdresser Customer Lecturer Scientist TV host Call agency representative Adventurer	Running Drinking coffee Playing tennis Watching football Socialising with friends Eating out Sleeping Using the phone Gossiping
Women	13.5%	Mother	Hugging a child

## Discussion

This study investigated the (mis) representation of women in the Mega Goal series in use at secondary schools in Saudi Arabia in 2023. The findings demonstrate that, despite the efforts to empower Saudi Arabian women laid down in the Saudi Vision 2030 and achievement of many of these targets such as appointing a number of women as ambassadors, ministers, directors and government consultors, women are still misrepresented and underrepresented in the EFL textbooks that are currently being used in the country’s secondary schools. This has far-reaching ramifications and therefore, course correction is essential to fully realize the goals of Vision 2030. For one, Moore (2007) argues that misrepresentation of both men and women in favour for one over the other could lead to an inaccurate understanding of the cultural and the social norms of a society. Such an inaccurate and misrepresentation of Saudi women is unwarranted as spurred by the Saudi 2030 Vision, Saudi women have been excelling in their careers. They have been occupying senior positions such as ambassadors, ministers, and astronauts, whereas the societal role of women found in influential ELF textbooks is reduced to a minimal presence and to simple activities and occupations, or mostly home-bound. Moreover, this misrepresentation and underrepresentation of Saudi women in the EFL teaching and learning context could potentially lead not only to an inaccurate picture of the cultural and social norms of Saudi Arabia as it aspires to empower women but could also run the risk of imprinting on Saudi students a negative image of women derived from the textbooks they study. Furthermore, the lack of visibility of empowered female role models in EFL textbooks could result in a stunting of female aspirations and a thwarting of the Vision 2030’s aims overall. As Gharbavi and Mousavi (2012) argue, imbalanced gender textbooks could leave a feeling of marginalisation, devaluation, and a sense of exclusion amongst female students, an outcome which hinders all the country’s efforts to empower women in Saudi Arabia.

In this way, the EFL textbooks currently in use in Saudi Arabia fall short in terms of presenting the equal opportunities for men and women in Saudi Arabia that are emphasised in the Saudi Vision 2030. Since its launch in 2016, the Vision has helped to bring about female empowerment in various fields in the country. However, the EFL textbooks which the country is currently using are failing to reflect such achievements.

## Conclusion

Social change refers to changes that are significant- that is, changes which alter the ‘underlying structure of an object or situation over a period of time’ (Giddens et al., 2005). Thus, social change does not include any and all changes, but only the big ones, changes which transform things fundamentally. Further, the ‘bigness’ of change is measured not only by how much change it brings about, but also, by the scale of the change, that is, by how large a section of society it affects, both intensively and extensively. Going by this definition, Saudi Arabia can claim social change with respect to the status of women seen in the evolution of ideas about their place in the society. This social change is reflective of the aims and objectives that Saudi Vision 2030 set out to achieve, especially where the emancipation and empowerment of women is concerned. However, a parallel cultural change still needs to be brought about as the culturally accepted and propagated picture of women leaves a great deal to be desired. This secondary change is much needed to change the aspirations and ambitions of the young people as they need to come to the adult world with a new mindset that does not inhibit the women from venturing into hitherto male bastions in the economic sphere. Needless to say, the pristine Arab culture and heritage need not feel threatened by these changes as the change has already been effected by the Vision 2030 developmental plan, it is the portrayal of women in textbooks which logically needs to be closer to the reality. Moreover, the change in the textbook portrayal is also needed as the ground facts concerning Saudi women’s economic contribution belie such under-representation. As per researchers at Brookings Institution, an American think tank, between 2021 and 2023, the participation of Saudi women in the workforce jumped 64%, and in some specialized sector such as hospitality, women now represent 40% of the workforce.

### Recommendations

Greater representation of women in a much wider field of occupations and social and daily activities must be included in future editions of EFL textbooks not only in order to comply with the Saudi Vision 2030, but also to encourage the ambitions of today’s young females to soar both upwards and outwards, so that the country’s future growth as envisioned in the Vision can be developed and sustained through their contributions to achieving their own aspirations and the development of the role of women in Saudi society overall.

## Author’s note

This work is dedicated to the strong women of Saudi Arabia who have shown the world their strength, courage, and determination. It is also dedicated to the inspiring women in my life: my mother Norah, my wife Marwa, and my two beautiful angels: Layla and Norah.

## Data availability statement

The raw data supporting the conclusions of this article will be made available upon request from the author.

## Author contributions

MA: Writing – original draft.
